# Analysis, Isolation, and Activation of Antigen-Specific CD4^+^ and CD8^+^ T Cells by Soluble MHC-Peptide Complexes

**DOI:** 10.3389/fimmu.2013.00218

**Published:** 2013-07-30

**Authors:** Julien Schmidt, Danijel Dojcinovic, Philippe Guillaume, Immanuel Luescher

**Affiliations:** ^1^Ludwig Center, University of Lausanne, Epalinges, Switzerland; ^2^TCMetrix Sàrl, Epalinges, Switzerland

**Keywords:** T cells, T cell receptor, tetramers, MHC, flow cytometry, coreceptor

## Abstract

T cells constitute the core of adaptive cellular immunity and protect higher organisms against pathogen infections and cancer. Monitoring of disease progression as well as prophylactic or therapeutic vaccines and immunotherapies call for conclusive detection, analysis, and sorting of antigen-specific T cells. This is possible by means of soluble recombinant ligands for T cells, i.e., MHC class I-peptide (pMHC I) complexes for CD8^+^ T cells and MHC class II-peptide (pMHC II) complexes for CD4^+^ T cells and flow cytometry. Here we review major developments in the development of pMHC staining reagents and their diverse applications and discuss perspectives of their use for basic and clinical investigations.

## Introduction

TCRαβ^+^ thymus derived T lymphocytes constitute the core of adaptive cellular immunity in higher organisms and play essential roles in their protection against pathogen infection and neoplastic transformations (1). CD8^+^ T cells differentiate to cytotoxic T lymphocytes (CTL) capable of efficient killing of cells displaying pMHC I complexes containing 8–10 residues long peptides, derived from a pathogen or cancer (2). CD8αβ coreceptor of CTL substantially enhances their antigen recognition, namely by strengthening the pMHC I ligand binding and bringing CD8-associated p56^lck^ to the TCR/CD3 complex, thus promoting TCR signal induction (3). In most cancer vaccines and immunotherapies, tumor antigen-specific CD8^+^ T cells take center stage (4, 5). Antigen-specific CD8^+^ T cells can be reliably detected, analyzed, and sorted by means of soluble pMHC oligomers (6, 7). Peptide-MHC complexes have limited life spans at 37°C and therefore MHC I molecules provide a dynamic display of endogenously produced peptides. pMHC complexes can be obtained by refolding of denatured heavy and light (β2m) chains in the presence of a peptide of interest (8). This procedure is simple, inexpensive, and yields highly pure pMHC complexes. Because the peptides bind to MHC I molecules in a canonical manner, in which both termini are firmly anchored in well-defined positions, these complexes are homogenous and structurally well-defined (9).

On the other hand, CD4^+^ T cells differentiate from a common precursor (TH0) in different types of effector cells, such as T help cells (Th1, Th2), or regulatory T cells (Treg). Efforts to detect antigen-specific CD4^+^ T cells by soluble pMHC II multimers fared less well than their class I counterparts for diverse reasons. MHC II molecules consist of non-covalently linked alpha and beta chains, both of which are transmembrane spanning proteins and their variable domains (alpha 1 and beta 1) form an open ended peptide binding groove that accommodates exogenous peptide usually 10–15 residues long (10). In contrast to MHC I, MHC II molecules, in particular HLA II molecules, are stable without a peptide, which allows loading of “empty” MHC II molecules with a peptide of interest (11). Newly synthesized MHC II molecules associate with invariant chain (li), which assists their refolding, prevents binding of endogenous peptides in the endoplasmic reticulum, and directs them to the endosomal/lysosomal pathway where they are loaded with peptides generated from exogenous antigens by lysosomal proteolytic activity (12). To prepare soluble recombinant MHC II molecules, their transmembrane and cytoplasmic domains are replaced by leucine zippers to prevent dissociation of the alpha and beta chains (13). With a few exceptions pMHC II complexes cannot be obtained in good yields by refolding and/or by mammalian expressions systems, but can be well expressed by insect expression systems, such as Drosophila S2 cells or SF9 cells infected with baculo virus (14, 15). While binding of soluble pMHC I complexes to CD8^+^ T cells is greatly strengthened by CD8, CD4 does not stabilize pMHC II binding to CD4^+^ T cells (16). Together with the fact that CD4^+^ T cells usually express lower affinity than CD8^+^ T cells, this accounts for frequent poor pMHC II tetramer staining and missing of a substantial fraction of antigen-specific CD4^+^ T cells; this is often aggravated by poor reagent quality, namely incomplete or disperse peptide loading (17).

## Chronological Overview of Soluble pMHC Class I Complexes and Their Use for T Cell Analysis

**Figure 1:**
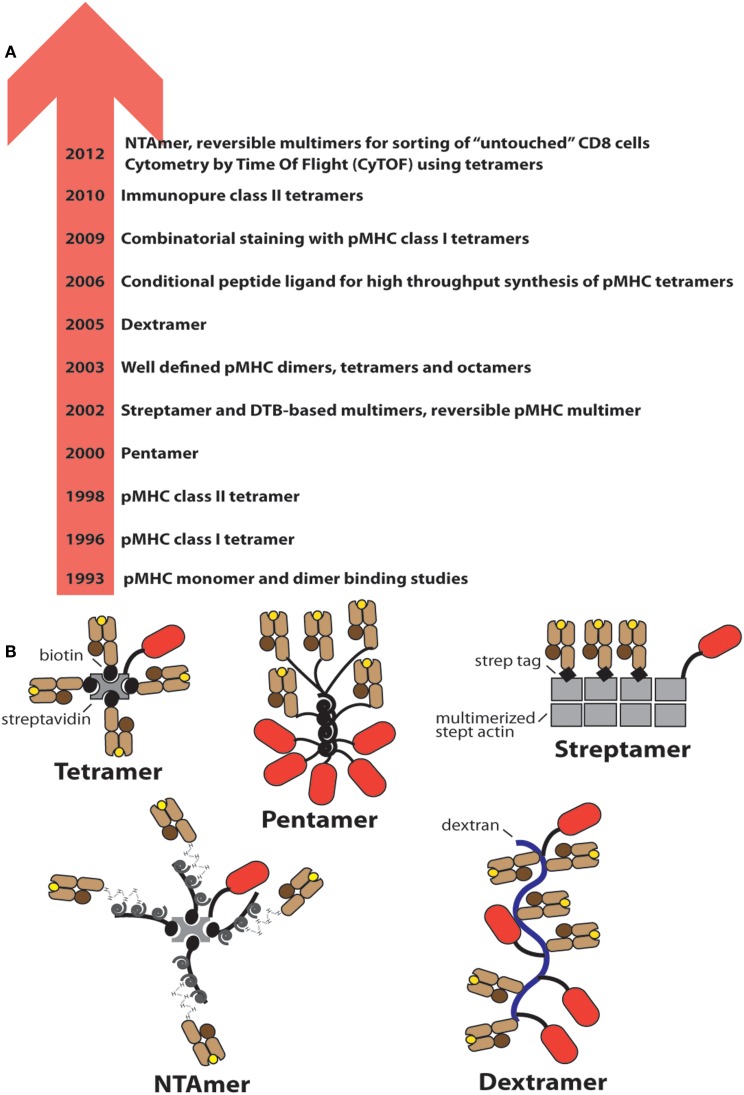
**Evolution of soluble pMHC complexes and their applications for T cell analysis and sorting**. **(A)** Chronological listing of milestones in the use of pMHC complexes for the detection and analysis of antigen-specific CD8^+^ and CD4^+^ T cells. **(B)** Cartoons of the most frequently used pMHC oligomers; the red oval represents PE.

Soluble recombinant pMHC complexes are valuable tools for investigations, analysis, and isolation of antigen-specific T cells (Figure 1). The smallest pMHC complex is a monomer, which according to SPR studies binds recombinant TCR with dissociation constants (*K*_D_) in the range of 1–1000 μM, and dissociation kinetic rates in the range of 1–1500 s (18), which precludes analysis by flow cytometry or fluorescence microscopy. Nevertheless, the binding and dissociation of monomeric pMHC complexes was extensively studied on CD8^+^ CTL by means of TCR photoaffinity labeling (19, 20). These studies revealed that coordinate binding of CD8 to TCR-associated pMHC I complexes greatly strengthens pMHC I binding, mainly by decreasing the TCR–pMHC dissociation (3). It is noteworthy that although pMHC binding data measured by SPR are markedly different than those measured on living CD8^+^ T cells, they correlate well with CD8^+^ T cell activation. The likely explanation for this is that intercellular TCR–pMHC interactions precede CD8 co-engagement, i.e., CD8 is recruited to sufficiently stable TCR–pMHC complexes (21).

Also in 1993 pMHC I dimers were described, with the capacity to fully activate CD8^+^ CTL clones, whereas monomers were inactive (22). The ability of pMHC I and later also pMHC II dimers to inhibit antigen-specific T cells *in vitro* and *in vivo* was shown to be a promising means to block autoimmunity (23, 24). Peptide-MHC dimers built on chemical linkers were described only many years later (see below).

In 1996 the first pMHC I “tetramer” was described and its usefulness demonstrated for the detection and analysis of virus-specific CD8^+^ T cells (25). The subunit pMHC I monomers carry a biotinylation sequence peptide (BSP) C-terminal of the alpha3 domain that is amenable to biotinylation with the biotin transferase BirA (26). Reaction of biotinylated pMHC I monomers with phycoerythrin-streptavidin (PE-SA) (or allophycocyanin-SA) typically results in mixtures of pMHC I conjugates of different stoichiometry and relative orientations and hence are better termed multimers (27). Our analysis indicated that such multimers contained species carrying up to 12 pMHC I monomer (unpublished results). Real tetramers can be prepared by using “tetra-grade” PE-SA and exhibit substantially reduced staining (see below).

In 1998 the first pMHC II multimers were described (28), which performed less well than their pMHC I counterparts, often exhibiting erratic, unreliable staining performance and according to one study approximately half of all antigen-specific CD4^+^ T cells, especially self-antigens-specific ones, are not detected by conventional multimers (29). Only in 2010 molecular defined pMHC II multimers were described and their superior staining performance demonstrated (30, 31) (Figure 1).

In 2000 “Pentamers” (by ProImmune) were introduced that comprise five pMHC I complexes and five PE. Despite a lower valency compared to conventional multimers, the staining performance of both reagents seems to be similar (32). In 2002 meeting the increasing need for sorting of antigen-specific CD8^+^ T cells namely for *in vitro* studies or adoptive cell transfer experiments, reversible multimers, based on biotin analogs, were introduced, which allow sorting of untouched CD8^+^ T cells (33, 34). A third type of reversible multimers, the NTAmers, based on multivalent Ni^2+^-NTA-His-tag complexes, followed nearly a decade later (35). In 2003 well-defined homologous pMHC dimers, tetramers, and octamers were described that contained linkers of defined lengths and configurations (36, 37). Binding and activation studies on cloned murine CTL and on CD8^+^ T cells from melanoma patients revealed that the binding avidity and CD8^+^ T cell activation critically depends not only on the valence, but also on pMHC–pMHC distances and configurations (38). In 2005 Dextramers (by Immudex) were introduced, which consist of long dextran fibers conjugated with multiple PE and pMHC I molecules (39) (Figure 1). While these studies contributed to improve analysis of antigen-specific T cells by means of pMHC oligomers, only recent developments have substantially increased their usefulness for comprehensive immune monitoring. For example an original new strategy allowing production of a wealth of different pMHC I complexes was introduced in 2006, i.e., the UV irradiation induced peptide exchange on pMHC I complexes containing a photoreactive peptide (40). This technique is useful especially for combinatorial multimer staining, independently established by two groups in 2009 (41, 42). Combinatorial multimer staining allows parallel detection of multiple specificities, which provides more information on precious, often small blood samples. One strategy uses binary color coding, i.e., antigen-specificity is defined by a unique combination of two colors (41), whereas the other strategy uses all possible color combinations, which affords higher number of color combinations, but also stronger reduction in fluorescence intensity (42). Three years later CyTOF was introduced, i.e., cytometry combined by time of flight measurements (43). The cells are labeled with lanthanide labeled pMHC multimers, resolved in a cytometer, converted in plasma, and its lanthanide content analyzed by mass spectrometry. Presently about 30 channels, i.e., different mass tags, are available, which provides more possibilities for parallel detection than flow cytometry (44). In 2012 also NTAmers were described, which are pMHC multimers built on NTA-Ni^2+^-His-tag interactions that are nearly as stable as biotin–SA bonds, but upon addition of imidazole instantly decay in their constituents. By using Cy5 labeled pMHC molecules, NTAmers allow measurements of pMHC monomer dissociation kinetics on CD8^+^ T cells (Figure 4).

## Well-Defined Soluble pMHC Complexes: Tools to Study CD8^+^ T Cells Ligand Binding and Activation

**Figure 2:**
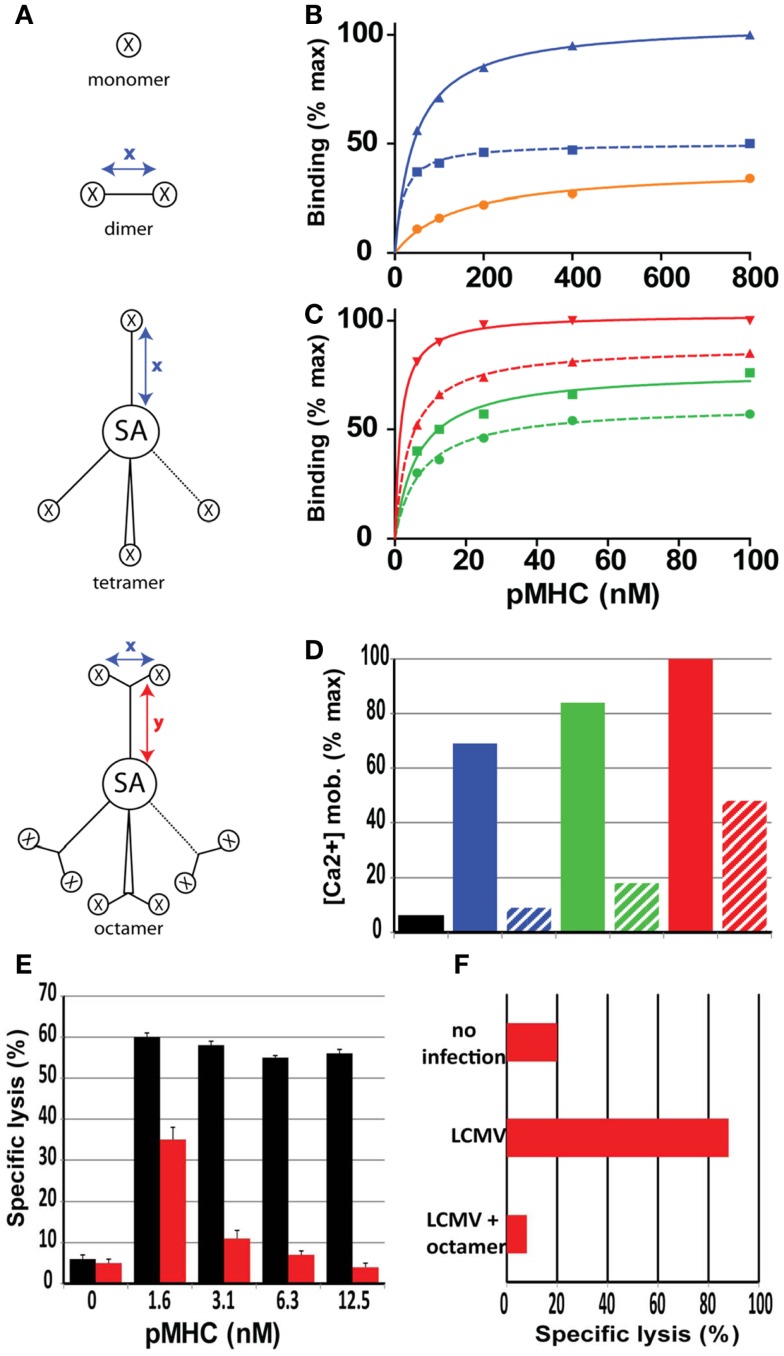
**Role of valence and spacer distances of soluble pMHC complexes on binding and activation of CD8^+^ T cells**. **(A)** Cartoons of mono, di, tetra, and octamer pMHC complexes, in which *x* and *y* indicate maximal spacer distances in Å, X a pMHC monomer and SA streptavidin. The spacer distances for the pMHC dimers varies from 18 to 90 Å. **(B)** Relative binding on CD8^+^ T cells of monomer (black), short dimer (blue), and long dimer (dotted blue). **(C)** Same as **(B)** with short tetramer (green), long tetramer (dotted green), short octamer (red), and long octamer (dotted red). Short distances are<20 Å and long distances are>80 Å. **(D)** Relative activation (Ca^2+^ mobilization) of CD8^+^ T cells after binding of the represented pMHC complexes. **(E)** Specific lysis of target cells (GP33 peptide sensitized P815/Db cells) was assessed following incubation with LCMV d8 CD8^+^ CTL in the presence of Db/GP33 monomer (black bars) or Db/GP33 long octamer (red bars). **(F)** Specific lysis of GP33 peptide sensitized P815/Db cells was assessed in LCMV infected mice that were or were not (LCMV) injected previously with octamer. Presented data were derived from Ref. (36–38).

While most soluble pMHC complexes used for detection and isolation of antigen-specific T cells are heterogeneous, complexes of defined valence, configuration, and pMHC–pMHC distances were made and tested on CD8^+^ T cells, which provided valuable insights on how they bind and are activated by pMHC complexes (Figure 2).

### pMHC I monomers

Due to the weak and transient nature of pMHC monomer binding to CD8^+^ (and CD4^+^) T cells, on cells binding studies require special techniques, such as TCR photoaffinity labeling (3, 19, 45, 46). To this end CTL clones were derived from mice immunized with a photoreactive derivative of the malaria circum sporozoite peptide PbCS_252–260_ [SYIPSAEK(ABA)I, ABA: 4-azido-benzoic acid] that specifically recognize this peptide derivative. These binding studies on living CD8^+^ T cells revealed that the coreceptor CD8αβ strongly strengthens TCR–pMHC interaction by coordinate binding to TCR-associated pMHC molecules (3). This increase in binding avidity varies in the range of 4- to 20-fold, depending of the clone and temperature and was mainly accounted for by decreased TCR–pMHC dissociation kinetics (45–47). The kinetics of pMHC monomer binding on living CTL exhibited a prominent temperature dependence, namely a dramatic decreases of dissociation rates with decreasing temperatures and biphasic association binding kinetics at 37°C, but not in the cold (3). Soluble monomeric complexes do not activate CD8^+^ T cells unless these are adhered to a substrate; however, it was demonstrated that peptides of soluble pMHC monomers can be transferred to CD8^+^ T cells CTL-associated MHC I molecules by an unknown mechanism and that this can engender CTL–CTL interactions (48, 49).

### pMHC dimers

The first pMHC dimers described were fusion proteins consisting of pMHC monomers fused to IgG and provided initial new insights in pMHC dimer binding to CD8^+^ T cells, namely that naïve, effector, and memory T cells exhibits substantially different binding and activation/inactivation properties (24, 50). More recently soluble pMHC I and II dimers were prepared that contain synthetic linkers of different length and flexibility (37). Testing of homologous K^d^/PbCS(ABA) dimers containing linkers of 12–130 Å showed that the binding avidity (*K*_D_) and the maximal binding (*B*_max_) were maximal for very short cross-linkers and gradually decreased with the spacer length of the linker to nearly 50% reduced *K*_D_ and *B*_max_ values at ≥80 Å (37) (Figure 2B). Dimers containing short flexible linkers very strongly activated CTL, which frequently induced CTL death via irreversible mitochondrial damages (38) (Figure 2D). By contrast dimers containing>80 Å long, rigid linkers induced no significant CTL activation and were able to inhibit CTL activation (36). Moreover, pMHC I (and II) mixed dimers were used to investigate the role of endogenous pMHC complexes for T cell activation. Antigen-specific T cell activation can be triggered by just a few cognate complexes, but for this high sensitivity, endogenous complexes are needed that although unable to trigger T cells by themselves, can substantially boost T cell activation by a few cognate pMHC complexes (51, 52). Conceptually it was proposed that such mixed pMHC dimers, or by analogy vicinal pMHC complexes on antigen presenting cells, promote coreceptor/lck association with TCR/CD3 and/or promote initial TCR/coreceptor co-aggregation on which T cell activation relies (53).

### pMHC tetramers and octamers

Conventional “tetramers” obtained by reacting biotinylated pMHC monomers with PE (or APC) labeled streptavidin are heterogenous, and unable to dissociate completely from CD8^+^ T cells (54). True tetrameric pMHC complexes can be prepared based on tetra-grade PE-SA or SA labeled with low molecular weight fluorochromes, e.g., Cy dyes. Real tetramers typically exhibit substantially lower binding avidities and fluorescence intensities, even when PE is used as in the conventional multimers. Moreover, as noted for pMHC dimers, tetramers containing short flexible linkers connecting pMHC to SA avidly bound to cloned CTL and vigorously activated these, whereas those containing long (>80 Å) rigid linkers bound less avidly, barely activated CTL and were able to antagonize CTL activation (36) (Figures 2C,D).

Finally octameric complexes were prepared by reacting Cy5-SA with Y shaped dimers in which two pMHC complexes were connected via short flexible or long rigid linkers to a biotin containing stem (36). Octamers composed of dimers with short inter pMHC distances avidly bound to and activated cloned CTL, whereas those consisting of dimers containing long rigid linkers bound less avidly and barely activated CTL. Owing to their high valence even octamers containing long rigid linkers exhibited avid binding to CTL and were able to efficiently inhibit CTL killing *in vitro* and *in vivo* and hence constitute a novel type of CD8^+^ T cell antagonists (36) (Figures 2C–F). Moreover, comparative binding studies on Melan-A-specific CD8^+^ T cell clones derived from a melanoma patient with A2/Melan-A dimers, tetramers, octamers containing linkers of different length and flexibility revealed striking differences depending on the state of CD8^+^ T cell differentiation (55). In particular dimeric complexes containing a short linker avidly and selectively stained a population of incompletely differentiation, dysfunctional CD8^+^ T cells, that are typically observed in advanced melanoma patients, but never in healthy individuals (55).

## Soluble pMHC Complexes Built on Ni^2+^-NTA–His-tag Chelate Complexes

### The make and nature of reversible pMHC complexes

**Figure 3:**
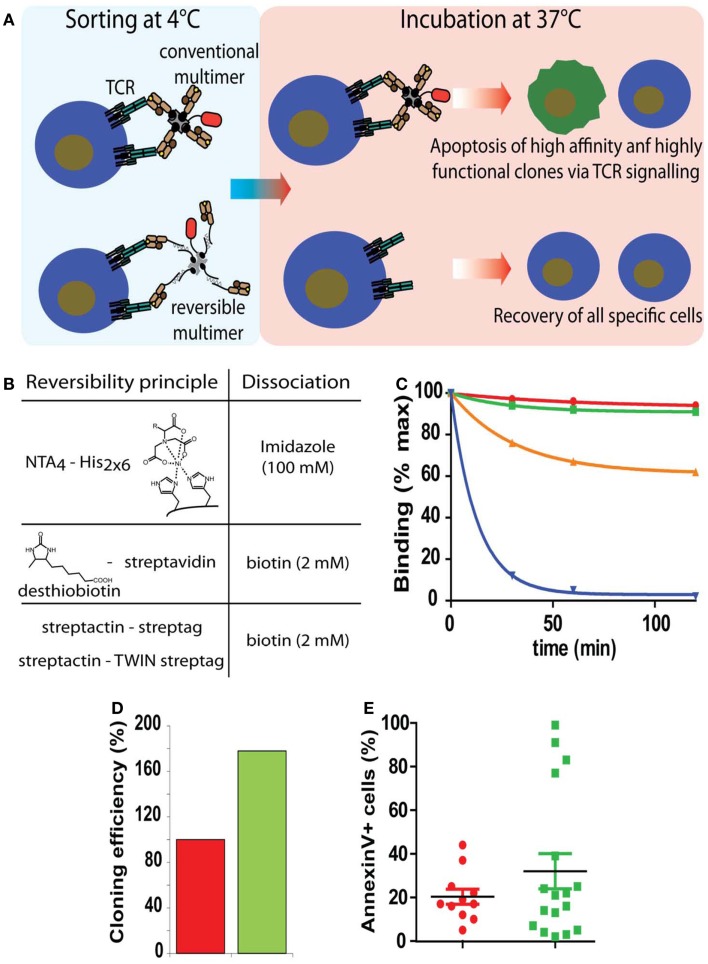
**Sorting of antigen-specific T cells with reversible pMHC multimers**. **(A)** Cartoon illustrating the principle of sorting of “untouched” T cells. After cell staining and sorting, reversible, but not conventional multimers can be removed and the T cells cultured at 37°C in the absence of potentially harmful pMHC complexes. **(B)** Representation of the three types of reversible bonds used for the preparation of pMHC oligomers and agent used to provoke dissociation. **(C)** Flu matrix_58–66_ peptide stimulated PBMC were stained at 4°C with A2/Flu multimers containing conventional irreversible (red), NTA_4_-His (green), streptactin-streptag (orange), or desthiobiotin–streptavidin (blue) scaffolds, washed and cell-associated fluorescence measured after different periods of incubation at 20°C. **(D)** Alternatively cells labeled with BSP (red) or NTAmer were FACS sorted and cloned by limiting dilution. Hundred percent refers to the number of clones obtained from BSP multimer sorted cells. **(E)** Randomly chosen clones were incubated with BSP multimer at 37°C, stained with AnnexinV, and analyzed by flow cytometry. Presented data were derived from (35).

Conventional BSP multimers are prepared by using the biotin–streptavidin irreversible tandem. Although for some applications this irreversible binding is desirable, for T cell staining it is not as multimers can bind and strongly activate those cells, leading sometimes to their death (Figure 3A). Nitrilotriacetic acid (NTA) at neutral aqueous solutions chelates bivalent cations, e.g., Ni^2+^, which in turn binds vicinal side chains of histidines. While the monomeric interaction is in the micro-molar range, picomolar dissociation constants were obtained for linear tetra-NTA compounds binding to pMHC molecules containing a C-terminal tandem His-tag (2xHis_6_) (35). While these coordination complexes are stable, also at elevated temperatures, they decay within a few seconds upon addition of free imidazole.

### Sorting of antigen-specific T cells by reversible soluble pMHC complexes

Although CTL are highly cytotoxic cells, they are remarkably susceptible to cell death, which can be induced by soluble pMHC complexes, e.g., directly by Fas/FasL mediated cytotoxicity, mitochondrial damages due to excessive CTL activation, or indirectly by fratricide caused by transfer of peptide from soluble to CTL-associated MHC molecules (37, 56). To prevent this and functional alterations, three types of reversible pMHC multimers have been described (Figure 3B) that allow sorting of “untouched” antigen-specific CD8^+^ (and CD4^+^) T cells (33–35, 57). Because T cell activation and signaling takes place at physiological temperatures, but not in the cold and pMHC monomers rapidly dissociate from T cells, they are stained with the multimers and sorted by FACS or MACS in the cold and cell-associated reagents are removed before culturing the sorted cells at 37°C. Two reversible multimers are built on altered biotin–SA scaffolds (33, 34, 57). The first ones, called streptamers, which are commercially available from IBA Life Science, consist of pMHC complexes containing C-terminal one or two streptags, i.e., a linear peptide sequence that mimics biotin and fluorescence labeled streptactin, a SA mutant that more avidly binds sterptags than SA. Because the binding of one streptag to streptactin is weak (micromolar range), it is advantageous to use a tandem streptag for improved stability. While streptamers (containing two streptags) in the cold are stable, decay becomes appreciable at 20°C, which risks to affect their shelf life, especially when brief periods of warming up cannot be excluded. The other type of reversible multimer contains desthiobiotin (DTB) in place of biotin, a biotin derivative with greatly weakened binding to SA (33). As the streptamer, these multimers can be readily dissociated by addition of free biotin. These reagents are only stable in the cold and readily decay at elevated temperatures, which can be reduced when using two DTB per pMHC entity. A third type of reversible pMHC multimers are NTAmer which being built on Ni^2+^-NTA–His-tag chelates complexes, rapidly decay upon addition of free imidazole (35). NTAmers built on NTA_4_-Ni^2+^-tandem His_6_-tag are nearly as stable as biotin–SA multimers and therefore are by far more stable than the other reversible multimers, especially at elevated temperatures (Figure 3C). As described below, multimer staining of antigen-specific CD4^+^ T cells is greatly improved at elevated temperatures (18°C) and therefore for their sorting NTAmers are recommended.

Sorting of antigen-specific CD8^+^ CTL with Streptamers or NTAmers yielded fully functional CTL, whereas when conventional tetramers were used their cytotoxicity and proliferation was impaired (35, 57, 58). The cloning efficiency of NTAmer sorted CTL was approximately twofold higher as compared to cloning with conventional, irreversible multimers. Analysis of CTL clones revealed that those preferentially lost by sorting with conventional multimers exhibited high physical and functional avidity, which is adverse in view of studies showing that high avidity CTL mediate superior tumor control (59) (Figures 3D,E). The use of reversible multimers thus is strongly recommended especially for sorting of antigen-specific T cells used in immunotherapy.

### Additional applications for NTA-based reversible scaffolds

#### PE-NTAmers allow detection of low avidity antigen-specific T cells

**Figure 4:**
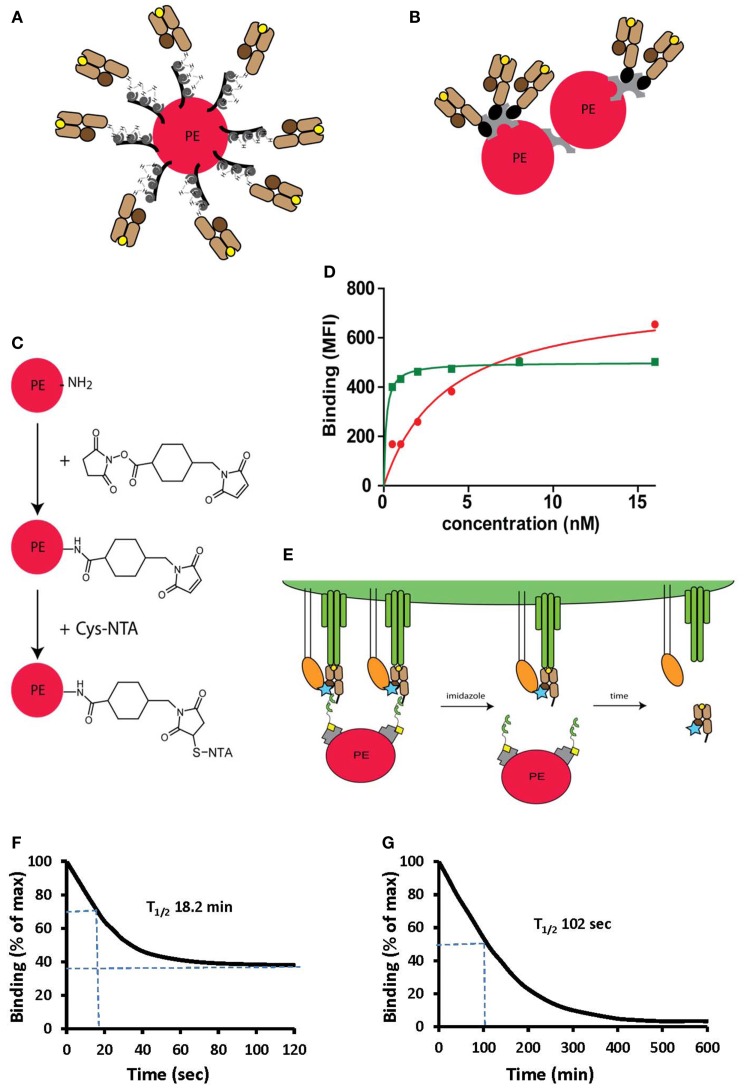
**Peptide-MHC oligomers built on switchable Ni^2+^-NTA-His-tag chelate complexes**. **(A,B)** Cartoons illustrating conventional BSP multimers pMHC **(B)** and reversible monomers directly coupled on PE via the NTA strategy **(A)**, where PE designates phycoerythrin. **(C)** The structure of a phycoerythrin molecule is shown in which lysine side chains reacted with a maleimido-*N*-hydroxysuccinimide ester and the resulting maleimido-PE will subsequently reacted with thio-NTA derivative. **(D)** Room temperature isotherms were shown for HLA-A2-Flu matrix_58–66_ BSP multimers (red circles) or PE-NTAmer (green squares) as assessed on Flu peptide stimulated PBMC. **(E)** Cartoon illustrating the two-step dissociation of PE labeled pMHC NTAmer from T cells. Imidazole induced rapid decay of cell-associated NTAmers and disappearance of PE fluorescence, followed by slower dissociation of Cy5 labeled blue monomers from the cells. **(F)** Cloned Flu matrix-specific CD8^+^ T cells were stained in the cold with conventional A2/Flu_58–66_ PE multimers, washed and cell-associated PE fluorescence measured by flow cytometry after the indicated periods of time. **(G)** Alternatively the cells were labeled with PE-NTAmers containing Cy5 conjugated A2/Flu_58–66_ monomers and the monomer dissociation from the cells was assessed by flow cytometry as illustrated in **(E)**. The inserted numbers indicate the dissociation half-lives *T*_1/2_. Presented data were derived from (33) and (35).

Previous pMHC binding studies on CD8^+^ T cells have revealed that the binding avidity substantially increases at decreasing pMHC–pMHC distances. In SA (and avidin derivatives) pMHC built complexes the minimal inter distance is in excess of the distance of their biotin binding sites (about 30 Å). By means of site-specific alkylation, we conjugated PE with linear NTA peptides and reacted this derivative with tandem His-tag carrying pMHC complexes [(35) and unpublished data, Figures 4A–C]. Phycoerythrin, a saddle shaped molecule, about fourfold larger than SA and with an abundance of surface lysines that permit the preparation of PE–pMHC conjugates with smaller pMHC–pMHC distances than in conventional multimers. The maximal degree of conjugation is 12–16 pMHC complexes per PE, but the maximal binding affinities were observed with conjugates containing 8–10 pMHC complexes per PE. Additionally these PE–pMHC NTAmers are molecularly better defined than conventional multimers, as they contain only one central PE, whereas conventional multimers contain higher order conjugates. In accordance with this, PE–pMHC NTAmer binding exhibit clear saturation and greatly increased binding avidity compared to conventional pMHC multimers and therefore can detect low avidity T cells that are missed by conventional staining reagents (Figure 4D). Conclusive detection of low avidity T cells is one of the formidable challenges in T cell monitoring, namely in cancer research where tumor antigen-specific T cells tend to be low avidity due to deletion of high affinity specificities by negative selection (60) and for CD4^+^ T cells, on which multimer binding is not strengthened by the coreceptor.

#### NTAmers allow pMHC monomer dissociation kinetic measurements

NTAmer are not only substantially more stable than other reversible pMHC multimers, but their induced dissociation is also more rapid and therefore can be used to measure accurate pMHC monomer dissociation kinetics on living CD8^+^ T cells (35). To this end NTAmers are prepared with Cy5 labeled monomers. CD8^+^ T cells are stained in the cold and the cell-associated PE and Cy5 fluorescences are measured before and after addition of imidazole (Figure 4E). After very rapid disintegration of the NTAmer, reflected by rapid disappearance of PE fluorescence, pMHC monomer dissociation from cells can be determined by from the decrease of cell-associated Cy5 fluorescence. While dissociation kinetics with pMHC multimers tend to be inaccurate due to incomplete dissociation and heterogenous composition, pMHC monomer dissociation reaches baseline and is more accurate (Figures 4F,G). This technique is less sensitive than SPR measurements, and inaccurate in the case of rapid kinetics, but provides information on pMHC–TCR interactions on cells, namely in the presence of CD8. Comparison of data obtained on a wide range of TCR-transduced cells, with NTAmers or directly by SPR showed a good correlation (unpublished data).

## Improved Detection and Analysis of Antigen-Specific CD4^+^ T Cells by Molecularly Defined MHC Class II Multimers

The performance of pMHC II multimers lags behind the one of pMHC I multimers, in particular they often fail to detect low avidity antigen-specific CD4^+^ T cells, which limits integral analysis of CD4^+^ T cell responses. Factor accounting for poor pMHC II multimer staining include (i) the coreceptor CD4, unlike CD8 fails to increase the pMHC binding avidity, (ii) certain types glycosylation and sialylation of CD4^+^ T cell molecules, (iii) lower affinity of MHC class II-peptide restricted TCRs, (iv) conformational diversity of pMHC complexes, and (v) poor quality of pMHC II multimers (61). While refolding of pMHC I complexes provides highly pure monomers, production of pMHC II monomers by loading of “empty” MHC II molecules with peptides of interest does not and therefore the corresponding pMHC multimers contain a reduced fraction of active monomeric complexes. The fraction of correctly loaded pMHC II monomers depends on the peptide and the MHC class II allele and can be well below 50% (62, 63). Tethering the peptide on the MHC II beta chain does not circumvent this caveat. Different strategies were described that allow isolation of molecularly defined pMHC II monomers. Peptide-MHC II tetramers containing defined fractions of cognate and non-cognate monomers were used to investigate the dependence of pMHC II tetramer staining of CD4^+^ T cells on pMHC II monomer purity. The efficiency of tetramer staining of antigen-specific CD4^+^ T cells dependents on the tetramer’s monomer purity; it decreases dramatically with the monomer purity for low avidity CD4^+^ T cells, but much less for high affinity cells. This explains why most successful pMHC II tetramer staining reported thus far concerns pathogen-specific CD4^+^ T cells and not tumor antigen-specific ones, which typically are of low avidity. The use of molecularly defined pMHC II tetramers permits conclusive analysis of low avidity CD4^+^ T cells (64).

### Peptide-MHC II production

Soluble recombinant MHC II molecules typically consist of alpha and beta chain where transmembrane and cytoplasmic domains of which have been replaced by acidic and basic leucine zippers (10). With very few exceptions soluble MHC II molecules are expressed in insect cells, either stably in *Drosophila* Schneider cells or transiently in sf9 cells using baculo virus (11, 61). Because in insect cells there is no MHC II-peptide loading apparatus, nor any retention mechanisms, they are typically secreted in “empty” form, i.e., without a nominal peptide cargo (12). These soluble MHC II molecules are purified usually be means of affinity chromatography using anti-MHC II antibody columns (65). Even though this provides highly pure MHC II proteins, the conditions used for elution, such as extreme pH, are prone to cause partial denaturation, which is an important factor for incomplete peptide loading of “empty” MHC molecules. In order to circumvent this, soluble pMHC molecules have been made containing a C-terminal His-tag, which allows gentle affinity purification at neutral pH on chelating columns, e.g., NTA or IDA columns. Loading of peptides of interest onto “empty” pMHC molecules is usually accomplished at reduced pH and elevated temperatures (e.g., pH 5.5–6, 30–37°C for 24–72 h) and is on faith, i.e., without knowledge of the extent of loading.

The peptide loading efficiency can be assessed by means of a peptide tag, usually an N-terminal one, such as His-tag, dinitrophenyl (DNP), or biotin (31, 66, 67). Such studies indicated that the degree of peptide loading can vary over a wide range and get as low as 10–20%. In addition peptide tags allow isolation of correctly loaded pMHC complexes, henceforth referred to as immunopure pMHC complexes (31). While this is attractive it renders the synthesis of pMHC class II multimers more tedious; importantly, the addition peptide tags may affect CD4^+^ T cell antigen recognition as suggested by observations that peptide truncations can have significant effects on CD4^+^ T cell antigen recognition (68–70). Therefore the use of conditional peptide tags would be desirable and could be prepared similarly as conditional peptide ligands, namely by linking it to the peptide via a photocleavable residue such as 2-[2-nitro-phenyl]-propionic acid (71, 72).

An alternative strategy to prepare soluble pMHC monomers is by tethering a peptide of interest to the N-terminus of the beta chain via a long and flexible linker (10). This method allows the preparation of murine pMHC class II monomers and tetramers, which are difficult to obtain via peptide loading, as “empty” mouse MHC II molecules are unstable. It is important to note that tethered on peptides may or may not be properly nested in the MHC II’s binding groove and that therefore the same concerns of denaturation, namely those related to immunoaffinity chromatography are applicable as mentions above. Moreover, it has been reported that depending on peptide truncations and linker length the tethered on peptides may bind in different registers and hence be differently recognized by antigen-specific CD4^+^ T cells (30, 62).

A major shortcoming of pMHC II tetramers is that they fail to stain a fraction of antigen-specific CD4^+^ T cells (29). This is especially a concern for tumor and self-antigen-specific CD4^+^ T cells, which tend to be of low avidity, due to deletion of high affinity cells by thymic negative selection and much less for pathogen-specific CD4^+^ T cells (29). This shortcoming curtails the usefulness of pMHC II tetramers especially in research related to cancer or autoimmunity (63, 73), because these fields of research become increasingly more important, we review in the following sections studies related to improving pMHC class II staining performance especially on low avidity CD4^+^ T cells.

### On the use of peptide tags to assess MHC II-peptide loading and to isolate immunopure monomers

**Figure 5:**
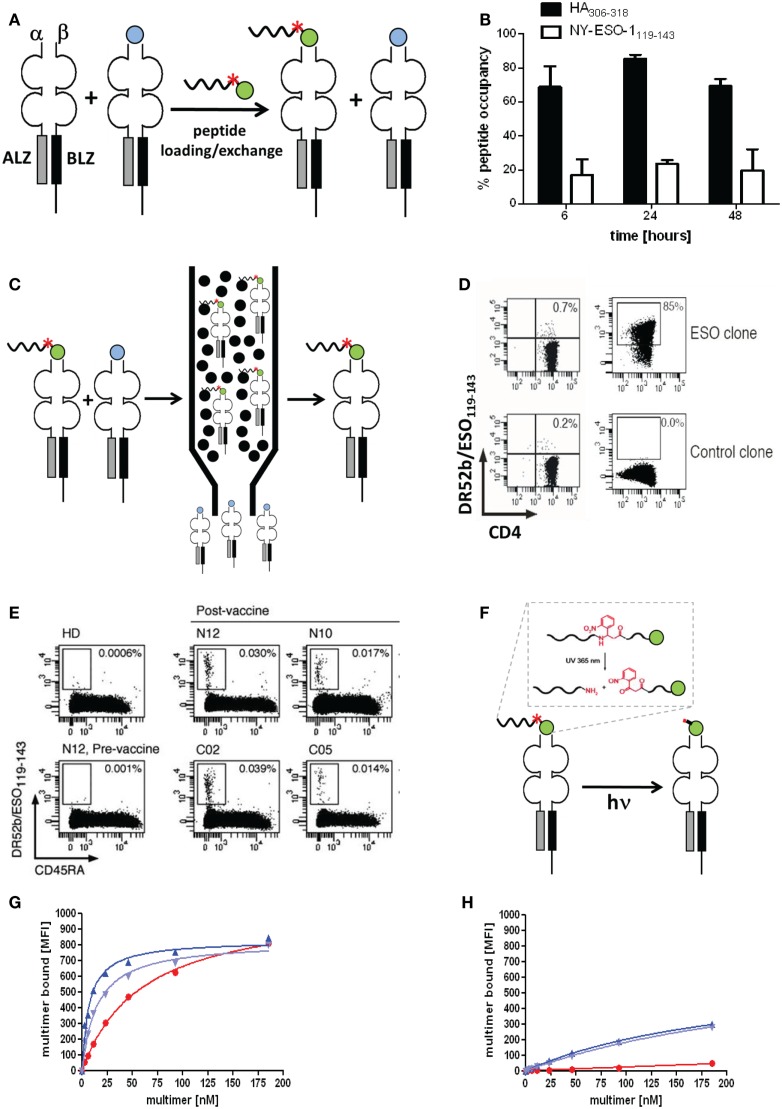
**Molecularly defined pMHC class II multimers detect low avidity CD4^+^ T cells**. **(A,C,F)** Cartoons illustrating the use of photocleavable (red asterisk) peptide tags to discriminate pMHC II complexes containing the peptide of interest (green ball) or irrelevant peptides (blue ball) **(A)**, to affinity purify correctly loaded complexes **(C)** and to remove the peptide tag, either His_6_ or desthiobiotin that is linked to the peptide via a β-nitrophenylglycine residue, which can be cleft by UV irradiation. **(B)** “Empty” HLA-DR4 was incubated for 6, 24, and 48 h at 37°C at pH 6, 32°C for 4 h with His_6_-HA_306–318_ (black bars) or His_6_-NY-ESO-1_119–143_ (white bars). Peptide occupancy was calculated as (amount of His_6_-peptide bound to the DR4)/(amount of total DR4). After gel-filtration the peptide occupancy was determined by ELISA using affinity-purified complexes as reference (100%). **(D)** Staining of DR52b/NY-ESO-1_119–143_-specific (top) or an irrelevant (SSX2-specific) CD4^+^ T cell clone (bottom) were stained with 5.6 nM of conventional (left) and “immunopure” DR52b/NY-ESO-1_119–143_ multimers (right) at 37°C for 1 h and analyzed by flow cytometry. **(E)** *Ex vivo* CD4^+^ T cells from PBMC of a DR52b^+^ healthy donors (HD) or of DR52b melanoma patients either pre- or post-NY-ESO-1 vaccination were stained as in E plus anti-CD45RA antibody and analyzed by flow cytometry. **(G,H)** The 37°C binding isotherms of “immunopure” DR4/HA_306–318_ (blue) and conventional multimers (red) on the DR4/HA_306–318_-specific clones 9 **(G)** or 8 **(H)**. The immunopure multimers contained [dark blue or not (light blue)] the N-terminal His_6_-tag that was used for affinity purification. Presented data were derived from (31).

In order to be able to measure peptide loading and to purify correctly loaded pMHC II complexes, several peptide tags have been described; for example: (1) An N-terminal dinitrophenyl (DNP) residue, allowing ELISA detection and isolation of DNP–pMHC complexes by means of anti-DNP mAb. This technique is expensive and addition of DNP renders poorly (Figure 5A) soluble peptide completely water insoluble (74). (2) N-terminal His_6_-peptide tags have been used that allow detection and isolation of specific pMHC complexes by means of chelators, such as Ni^2+^-NTA or IDA (31, 75). Advantages of this strategy include facile peptide synthesis and improved solubility of peptides and inexpensive availability of chelators for affinity chromatography and ELISA applications. Disadvantages include (i) losses due to inadequate binding affinity, (ii) backgrounds due to non-specific co-ligation, and (iii) incompatibility with multimer formation based on His-tag-NTA chelate complexes (35). (3) Biotin or desthiobiotin tag can be used for the detection of specifically peptide loaded pMHC II-peptide complexes. Disadvantages of these strategies include: (i) incompatibility with tetramer formation by means of biotin and streptavidin; (ii) for affinity purification of specially peptide loaded pMHC complexes, biotin binding tends to be too strong and DTB binding too weak (unpublished data); and (iii) aggravation of peptide solubility problems by addition of biotin.

Previous studies with His_6_ or Cy5.5 tagged peptides revealed that peptide loading to “empty” HLA-DR4 and DR52b molecules is remarkably variable. As shown in Figure 5B the viral Flu HA peptide bound to over 80% of “empty” DR4 molecules, whereas the tumor antigen NY-ESO-1 peptide bound to only 20%. Similar striking differences were observed for other viral and tumor antigen derived epitopes on “empty” DR4, DR52b, and DP4 molecules, with the viral peptides typically being the better binders. It may be speculated that viruses that are older than adaptive immunity of mammals, like the influenza virus, actively participated in the evolutionary shaping of the MHC locus.

By means of an N-terminal His_6_-peptide tag and IDA chromatography immunopure DR52b/NY-ESO-1_119–143_ monomers were produced and with these PE labeled multimers, which exhibited dramatically improved staining a cloned ESO-specific CD4^+^ T cells as compared to conventional DR52b/NY-ESO-1_119–143_ multimers (Figures 5A,C–E) (31). More importantly, these immunopure multimer allowed detection and detailed phenotypic and repertoire analysis of DR52b/NY-ESO-1_119–143_ specific CD4^+^ T cell in PBMC from melanoma patients post, but not pre vaccination with NY-ESO-1 protein. In view of numerous reports indicating that residues flanking the peptide binding core sequence can sensibly affect their interaction with MHC class II molecules, we established a procedure to remove the peptide tag after affinity purification. To this end we used photocleavable 2-[2-nitro-phenyl]-propionic acid to link the His_6_-peptide tag to the HA_306–318_ peptide, which allowed removing this tag by UV irradiation at nanometers (Figure 5F). The photocleavage is rapid (∼4 min using a 45 W mercury fluorescence lamp emitting at (365 ± 40 nm) and nearly quantitative (∼90%) (data not shown). We then used this technique to prepare immunopure DR4/HA_306–318_ multimers and compared their staining of cloned, flu-specific CD4^+^ T cells before and after removal of the His_6_-peptide tag. The multimer staining on one clone was modestly higher when the His_6_-tag was present, but was unaffected on another clone, indicating that this tag may affect pMHC II binding and/or recognition on some, but not other CD4^+^ T cell clones. What this experiment also shows is that the immunopure multimers bind significantly more avidly than the conventional multimer and that this difference is especially notable on low avidity clones, which are not significantly detected by the normal multimers (Figures 5G,H).

### Detection limits of pMHC II multimers and how they can be increased

A major shortcoming of pMHC II multimers is their failure to detect some antigen-specific CD4^+^ T cells. Studies using 2D binding assays it has been shown that a substantial fraction of antigen-specific CD4^+^ T cells responding in functional assays are not detected by tetramer staining, but are visible in a 2D binding assay (29). Interestingly comparison of CD4^+^ T cells specific for a self versus viral antigens showed that the proportion of tetramer undetectable CD4^+^ T cells is higher for the latter than the former. This is consistent with the view that for self-antigens CD4^+^ T cells expressing high affinity TCR are deleted by negative thymic selection (76). It is important to note that the vast majority of tumor antigens are self-antigens and that therefore tumor antigen-specific CD4^+^ (and CD8^+^) T cell responses tend to be in average of lower avidity than viral specific CD4^+^ T cells.

**Figure 6:**
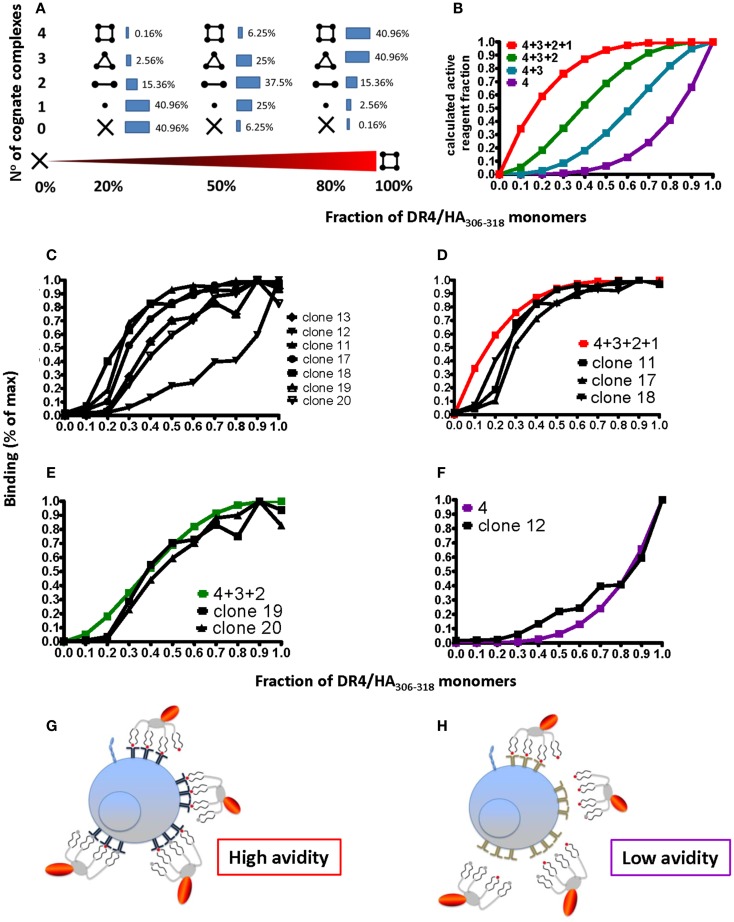
**Degree of peptide loading determines the avidity detection threshold on pMHC II multimer detection**. **(A,B)** Cognate (DR4/HA_306–318_) and irrelevant (DR4/CLIP) pMHC II monomers were mixed in different ratios (*x*-axis; fractions of one) and reacted with streptavidin–PE to make tetramers. For 0, 20, 50, 80, and 100% of cognate monomers the compositions of tetramers containing *X* = 0, 1, 2, 3, or 4 cognate monomers were calculated according to the binominal distribution and expressed in %, indicated by the inserted numbers **(A)**, with 100% being the sum of each row; alternatively it was plotted on the *y*-axis as fraction of 1 against the fraction of cognate/non-cognate complexes (*x*-axis) for the indicated four avidity levels T cell binding (red: all four complexes bind; green: three; blue: two; and purple: one). **(C)** Seven DR4/HA_306–318_-specific CD4^+^ T cell clones were incubated with 18.5 nM DR4 tetramers with DR4 tetramers containing the indicated factions of HA_306–318_ and CLIP peptides (*x*-axis; fraction of one) for 1 h at 37°C and cell-associated fluorescence assessed by flow cytometry and expressed in % of maximal binding (*y*-axis). **(D–F)** From these binding isotherms those observed on the clones 11, 17, and 18 match the high avidity binding curve (in red) **(D)**, those recorded on the clones 19 and 20, match the green curve **(E)** and the one measured on the clone 12 the purple curve **(F)**. **(G,H)** The cartoon representation of high **(G)** and low avidity **(H)** CD4^+^ T cells as defined by their ability to bind mixed tetramers.

While on CD8^+^ T cells pMHC binding is substantially strengthened by coordinate binding of CD8 to TCR-associated pMHC complexes, CD4 does not significantly contribute to pMHC binding (10). In addition, pMHC I complexes obtained by refolding all contain the peptide of interest; because this is not the case for pMHC II complexes obtained by loading of empty MHC II molecules, CD4^+^ T cell tetramer staining can be compromised by incomplete peptide loading. To investigate this, tetramers were prepared that contained defined proportions of immunopure cognate and non-cognate pMHC monomers. Using binominal distribution the average contents of cognate pMHC II monomer, dimer, trimer, and tetramer were calculated for different ratios of cognate/non-cognate complexes used to make the tetramer (Figure 6A); from these four different binding curves can be calculated for the scenarios that a tetramer can stain a CD4^+^ T cell when it contains (1) one, two, three, or four or (2) two, three, or four or (3) three and four or (4) only four cognate monomers (Figure 6B). Staining of seven randomly chosen DR4-restricted Flu HA_306–318_ specific CD4^+^ T cell clones (Figure 6C) with tetramers containing graded fractions of cognate, immunopure DR4-HA_306–318_ monomer, showed that such binding curves are found, i.e., that clones can be grouped in different discrete avidity ranges (Figures 6D–F). Importantly, this shows that low avidity CD4^+^ T cells will not be detected by tetramer staining, unless these contain only cognate pMHC monomers (Figure 6F). In view of the fact that pMHC II tetramer staining risks to miss a substantial fraction of antigen-specific CD4^+^ T cells, the use of immunopure tetramers is highly recommended in order to detect low avidity cells (Figures 6G,H).

The tetramer staining efficiency also critically depends on CD4^+^ T cells surface glycosylation and pretreatment of cells with a broadly active neuraminidase (e.g., from *Vibrio cholerae*) can increase tetramer staining by nearly 100% (77, 78). Testing on 37 Flu-specific Th1 clones we found increases of tetramer staining upon desialylation in the range of 20–95% (unpublished data).

## Conclusion

Wide ranges of soluble pMHC I-peptide oligomers have been prepared and used to analyze, activate, inactivate, and sort antigen-specific CD8^+^ T cells. Well-defined pMHC dimers, tetramers, and octamers containing linkers of defined length and flexibility were instrumental in dissecting mechanisms governing pMHC binding by CD8^+^ T cells and the ensuing cell activation and eventual cell death. Reversible multimeric pMHC complexes emerged as useful reagents to sort “untouched” CD8^+^ T cells; of particular interest were “switchable” NTAmer that additionally allowed measurement of pMHC monomer dissociation kinetics on living cells and detection of low avidity T cells. Detection of antigen-specific CD4^+^ T cells by pMHC II multimers overall was more challenging and prone to miss significant fractions of cells, namely low avidity CD4^+^ T cells; detection of these cells was dramatically improved by using molecularly defined multimers.

## Conflict of Interest Statement

The authors declare that the research was conducted in the absence of any commercial or financial relationships that could be construed as a potential conflict of interest.
